# Neuroimmunological effects of omega-3 fatty acids on migraine: a review

**DOI:** 10.3389/fneur.2024.1366372

**Published:** 2024-05-06

**Authors:** Ting-Bin Chen, Cheng-Chia Yang, I-Ju Tsai, Hao-Wen Yang, Yung-Chu Hsu, Ching-Mao Chang, Chun-Pai Yang

**Affiliations:** ^1^Department of Neurology, Neurological Institute, Taichung Veterans General Hospital, Taichung, Taiwan; ^2^Department of Healthcare Administration, Asia University, Taichung, Taiwan; ^3^Department of Neurology, Kuang Tien General Hospital, Taichung, Taiwan; ^4^Department of Medical Research, Kuang Tien General Hospital, Taichung, Taiwan; ^5^Management Office for Health Data, China Medical University Hospital, Taichung, Taiwan; ^6^Department of Family Medicine, Kuang Tien General Hospital, Taichung, Taiwan; ^7^Division of Neurology, Department of Internal Medicine, Ditmanson Medical Foundation ChiaYi Chistian Hospital, Chiayi, Taiwan; ^8^Center for Traditional Medicine, Taipei Veterans General Hospital, Taipei, Taiwan; ^9^Institute of Traditional Medicine, National Yang Ming Chiao Tung University, Taipei, Taiwan; ^10^School of Medicine, College of Medicine, National Yang Ming Chiao Tung University, Taipei, Taiwan; ^11^Ph.D. Program in Translational Medicine, National Chung Hsing University, Taichung, Taiwan

**Keywords:** eicosapentaenoic acid (EPA), docosahexaenoic acid (DHA), omega-3 fatty acids, neuroinflammation, neurogenic inflammation EPA

## Abstract

Migraine is a highly prevalent disease worldwide, imposing enormous clinical and economic burdens on individuals and societies. Current treatments exhibit limited efficacy and acceptability, highlighting the need for more effective and safety prophylactic approaches, including the use of nutraceuticals for migraine treatment. Migraine involves interactions within the central and peripheral nervous systems, with significant activation and sensitization of the trigeminovascular system (TVS) in pain generation and transmission. The condition is influenced by genetic predispositions and environmental factors, leading to altered sensory processing. The neuroinflammatory response is increasingly recognized as a key event underpinning the pathophysiology of migraine, involving a complex neuro-glio-vascular interplay. This interplay is partially mediated by neuropeptides such as calcitonin gene receptor peptide (CGRP), pituitary adenylate cyclase activating polypeptide (PACAP) and/or cortical spreading depression (CSD) and involves oxidative stress, mitochondrial dysfunction, nucleotide-binding domain-like receptor family pyrin domain containing-3 (NLRP3) inflammasome formation, activated microglia, and reactive astrocytes. Omega-3 polyunsaturated fatty acids (PUFAs), particularly eicosapentaenoic acid (EPA) and docosahexaenoic acid (DHA), crucial for the nervous system, mediate various physiological functions. Omega-3 PUFAs offer cardiovascular, neurological, and psychiatric benefits due to their potent anti-inflammatory, anti-nociceptive, antioxidant, and neuromodulatory properties, which modulate neuroinflammation, neurogenic inflammation, pain transmission, enhance mitochondrial stability, and mood regulation. Moreover, specialized pro-resolving mediators (SPMs), a class of PUFA-derived lipid mediators, regulate pro-inflammatory and resolution pathways, playing significant anti-inflammatory and neurological roles, which in turn may be beneficial in alleviating the symptomatology of migraine. Omega-3 PUFAs impact various neurobiological pathways and have demonstrated a lack of major adverse events, underscoring their multifaceted approach and safety in migraine management. Although not all omega-3 PUFAs trials have shown beneficial in reducing the symptomatology of migraine, further research is needed to fully establish their clinical efficacy and understand the precise molecular mechanisms underlying the effects of omega-3 PUFAs and PUFA-derived lipid mediators, SPMs on migraine pathophysiology and progression. This review highlights their potential in modulating brain functions, such as neuroimmunological effects, and suggests their promise as candidates for effective migraine prophylaxis.

## 1 Introduction

Migraine, a disorder influenced by genetics and characterized by multiple factors involving neurological, glial, and vascular systems, ranks as the second most debilitating condition worldwide, impacting the health of over one billion individuals globally (1). While there are established pharmacological guidelines for the prevention of migraines (2, 3), a significant number of patients still experience unmet needs, with many not benefiting from or failing to respond to preventative treatments (4, 5).

Migraine manifests as recurrent bouts of moderate to severe headache lasting between 4 to 72 h, typically one-sided, and accompanied by nausea, sensitivity to light (photophobia), sensitivity to sound (phonophobia), and/or vomiting (6, 7). Its underlying mechanisms are multifaceted, encompassing genetic variations, environmental influences, cerebral artery constriction, abnormalities in monoamine neurotransmitters, oxidative stress, mitochondrial disturbances, the involvement of calcitonin gene-related peptide (CGRP), neurogenic inflammation, and broader neuroinflammation (6). The once-prevailing vascular theory, which posited that headaches stem from dilation of dural and extracranial blood vessels, has been refuted, with such vasodilation now regarded as an effect rather than a cause of migraines (8). The prevailing theory suggests that migraine is a neurovascular disorder initiated within the central nervous system (CNS), characterized by an increased sensitivity of the trigeminovascular system (TVS) leading to pain. From a neurobiological standpoint, the development and intensification of migraine into a debilitating condition involve intricate interactions between neurons, glial cells, vascular components, and inflammatory mediators within the TVS (6, 9–12). The interactions within the TVS, immune cells, and ongoing neurogenic or neuroinflammatory responses play significant roles in migraine pathophysiology, potentially leading to the establishment of chronic maladaptive pain.

Omega-3 polyunsaturated fatty acids (PUFAs), such as eicosapentaenoic acid (EPA) and docosahexaenoic acid (DHA), are vital micronutrients for neurological health. These fatty acids play a key role in numerous physiological processes and have shown benefits in managing cardiovascular, neurological, and psychiatric conditions, demonstrating a well-tolerated side effect profile (13–15). Specifically, omega-3 PUFAs and their derivatives exhibit strong anti-inflammatory, anti-nociceptive, and neuromodulatory effects, impacting the pathophysiology of migraines (14, 16, 17). Moreover, various specialized pro-resolving mediators (SPMs) produced from omega-3 PUFAs help in moderating undue neuroinflammatory responses and inflammatory pain, facilitating communication between glial cells and neurons. This could significantly contribute to the mitigation of migraine symptoms (18). This paper aims to outline the neuroimmunological actions of omega-3 PUFAs in migraine prevention and offer an up-to-date review on this subject.

## 2 Different phases of migraine

Migraines can be categorized into five distinct phases: the prodrome, a possible aura, the headache attack, the postdrome (recovery stage), and the interictal phase. However, not all individuals experience every stage (19). The prodrome, marking the initial clinical phase of a migraine, manifests through symptoms believed to result from hypothalamic activation, such as difficulty concentrating, fatigue, yawning, neck stiffness, feelings of depression, and irritability (20, 21). In the lead-up to the migraine, alterations in the connectivity between the hypothalamus, brainstem, spinal trigeminal nuclei, and the dorsal rostral pons in the hindbrain have been noted as early as 48 h before the pain onset. Additionally, specific brainstem activations have been recorded 24 h before the headache begins (22, 23). These findings suggest that the genesis of migraine attacks is associated with intrinsic brain dysfunction in more central regions, including the diencephalic nuclei.

The aura phenomenon, representing a temporary dysfunction of the CNS, occurs in 25% of migraines (22, 23). Cortical spreading depression (CSD), characterized by a strong wave of neuronal depolarization accompanied by glial and vascular activation, is widely believed to underlie the aura phase of migraines (24). During CSD, an excitatory wave sweeps across the cerebral cortex, altering the cerebral blood supply, enhancing neuronal excitability, and triggering the release of neuroactive peptides. This results in focal hyperemia with increased tissue metabolism that persists for minutes and is succeeded by a wave of neuronal depolarization and significant oligemia (25). CSD has been demonstrated to activate the trigeminovascular system (TVS) both centrally and peripherally by exposing the dura to inflammatory molecules, indicating that susceptibility to CSD may contribute to the initiation of migraine attacks (26, 27). Furthermore, migraines with aura have been linked to neuroinflammation, as evidenced by preclinical models and imaging studies of the human migraine condition (28).

The headache phase follows the prodromal or aura stages of a migraine, marked by the activation and sensitization of the trigeminovascular system (TVS) and brainstem, leading to disrupted sensory processing and various neurological symptoms indicative of altered brain excitability (29). The activation of TVS pain pathways triggers the release of pro-inflammatory neuropeptides and neurotransmitters such as CGRP, substance P, PACAP, VIP, and NO. These molecules interact with receptors on meningeal blood vessels and the dura mater, causing vasodilation, activation of nociceptive fibers, and an inflammatory response (8, 12). CGRP, a potent vasodilator presents in both peripheral and central neurons, plays a crucial role in neurogenic inflammation and sensitization (30). CGRP released from cutaneous trigeminal nerve fibers and acts on surrounding Schwann cells, perpetuating cutaneous allodynia through a cyclic AMP-dependent mechanism that generates NO in endosomes (31). Inflammatory by-products and oxidative stress can stimulate CGRP receptors, indicating their role as detectors of environmental oxidative states (32). Signals from dural perivascular neurons instigate a sequence of events that lead to the release of inflammatory mediators, sterile meningeal inflammation, and sensitization of pain-processing brain regions (33). Afferent fibers from the trigeminal ganglion synapse on neurons in the trigeminal cervical complex (2), and ascending pathways transmit signals to various brain regions, causing pain, cutaneous allodynia, and sensory disturbances like photophobia and phonophobia (6, 34).

The transition from episodic to chronic migraine involves changes in nociceptive thresholds and central sensitization, driven by factors such as high frequency of migraine attacks, overuse of acute medications, obesity, stress, female sex, psychiatric conditions, and low socioeconomic status (34). Significant brain alterations in pain-processing areas, abnormal pain modulation, central sensitization, cortical hyperexcitability, neurogenic inflammation, and ongoing neuroinflammation are central to the development and persistence of chronic migraines (35). Collectively, these findings suggest that in individuals with a genetic predisposition, the pathophysiology of migraine is characterized by repeated activation and sensitization of trigeminal nerves by meningeal inflammation, influenced by significant brain activity and resulting in structural and functional brain changes (36).

## 3 Neurogenic inflammation in migraine

Neurogenic inflammation is defined as an acute sterile inflammation triggered by the release of neuropeptides from peripheral nociceptive fibers, leading to plasma protein extravasation and vasodilation (10). The relevance of dural neurogenic inflammation as an initiator and driver of acute migraine attacks has been proposed for decades. Neurogenic inflammatory reactions occur after activation of the TVS in the dura matter due to electrical, chemical stimulation, or the application of an inflammatory soap, rather than by immunological events or pathological microorganisms. Evidence has demonstrated that local sterile meningeal inflammation plays a role in inducing prolonged activation and sensitization of meningeal afferents (5). Neurogenic inflammation in acute migraine attacks is characterized by a TVS activation-induced release of neuropeptides, such as CGRP and substance P, from trigeminal nerve fibers at the dura, which causes increased vascular permeability, plasma protein extravasation, leukocyte infiltration, vasodilation, mast cell degranulation, activation of meningeal nociceptors, and the subsequent release of inflammatory mediators (5). Antidromic axon reflexes may then cause further release of CGRP in the dura (30). Prolonged TVS activation may result in a sustained release of CGRP. The continued activation of C-fibers during recurrent migraine attacks and the ensuing activation of CGRP-related nociception maintain TVS sensitization, thereby promoting migraine chronicity (5, 30).

Neurogenic inflammation refers to an acute, non-infectious inflammation initiated by neuropeptide release from peripheral pain-sensing fibers, causing plasma protein leakage and blood vessel expansion (10). The concept of neurogenic inflammation within the dura mater serving as a catalyst and perpetuator of acute migraine episodes has been considered for many years. This type of inflammation is triggered in the dura mater by activation of the trigeminovascular system (TVS) through electrical or chemical stimuli, or the application of an inflammatory substance, rather than immune or microbial factors. Research indicates that localized sterile inflammation in the meninges contributes to the prolonged activation and sensitization of meningeal sensory nerves (10). In the context of acute migraine, neurogenic inflammation involves the release of neuropeptides such as CGRP and substance P by trigeminal nerve endings within the dura mater. This release leads to increased vascular permeability, plasma protein extravasation, white blood cell infiltration, vasodilation, mast cell breakdown, stimulation of meningeal pain receptors, and further release of inflammatory substances (10). Additionally, antidromic axon reflexes could trigger additional CGRP release in the dura (33). Continuous TVS activation might cause an ongoing discharge of CGRP. The repetitive triggering of C-fibers during migraine attacks and the resultant stimulation of CGRP-mediated pain pathways keep the TVS in a state of heightened sensitivity, thus contributing to the progression of migraine into a chronic condition (10, 33).

## 4 Neuroinflammation in migraine

Neuroinflammation refers to an inflammatory reaction within the CNS, regulated by cytokines, chemokines, reactive oxygen species (ROS), and secondary messengers such as nitric oxide (NO) and prostaglandins (37). These compounds are generated by activated microglia — the CNS’s primary immune cells — and astrocytes, as well as by neurovascular units consisting of neurons, pericytes, and endothelial cells (38). Neuroinflammatory mechanisms have been implicated in both episodic and chronic forms of migraine, regardless of the presence of aura (10).

### 4.1 Neuron–glia communication

Within the trigeminal ganglion, neurons and surrounding satellite glial cells engage in mutual communication during activation of the TVS. CGRP, when released from the bodies or axonal swellings of stimulated trigeminal neurons, can trigger satellite glial cells within the trigeminal ganglia, as well as microglia and astrocytes in the trigeminal nucleus caudalis. This interaction leads to the glial cells producing inflammatory cytokines and NO, which in turn further stimulates trigeminal neurons (10, 39, 40). The presence of cytokines and NO increases CGRP release, forming a self-reinforcing loop within the ganglion. Additionally, direct neuron–glia communication is facilitated through two-way calcium signaling, primarily via purinergic P2 receptors (notably P2X7) and gap junctions (39). This intricate neuron–glia dialogue intensifies pain signaling and contributes to central sensitization (41, 42).

### 4.2 Immunological effect of CSD

The consensus is that CSD triggers the trigeminovascular system, leading to a meningeal inflammatory response (43). CSD is characterized by a propagating wave of neuronal and glial depolarization driven by significant ion movements across the cortex, which subsequently results in prolonged suppression of neuronal activity. This mechanism can perpetuate the activation and sensitization of meningeal pain receptors through the release of protons, potassium ions, and glutamate. Such releases promote local neurogenic inflammation and activate the trigeminovascular system (10). The ensuing broad activation of ion pumps, coupled with elevated metabolism and oxygen demand, may cause brain tissue hypoxia.

Cellular oxygen deficiency hampers the mitochondrial electron transport chain, restricting or stopping mitochondrial oxidative phosphorylation. This leads to the accumulation of excess oxygen radicals, contributing to oxidative stress and further triggering CSD (44). Research has linked migraine to mitochondrial dysfunction, evidenced by reduced mitochondrial membrane potential and the dysregulated activity of mitochondrial permeability transition pores. This dysfunction results in neuronal energy depletion, cell death, reduced pain tolerance, and migraine episodes (45). Therefore, a combination of brain energy shortage, mitochondrial dysfunction, and oxidative stress surpassing the body’s antioxidant defenses is recognized as a key element in migraine pathophysiology (45, 46).

At the cellular level, CSD can incite neuroinflammation through a series of interactions involving the pannexin-1 channel, caspase-1, interleukin (IL) 1β, and high-mobility group box 1 in neurons. Additionally, there is nuclear factor-κB-dependent upregulation of inflammatory genes, such as cyclooxygenase and inducible nitric oxide synthase, in astrocytes. These mechanisms lead to the prolonged activation of trigeminal neurons, astrocytes, and microglia around meningeal blood vessels, inflammatory cascades within brain parenchyma reaching the glia limitans, and the dispersal of arachidonic acid (AA) and potassium ions into the perivascular area (9, 10, 39). Simultaneously, the assembly and activation of the NLRP3 (nucleotide-binding domain-like receptor family pyrin domain containing-3) inflammasome complex, regulated by NF-kB, are enhanced in neurons, microglia, and astrocytes within the trigeminal ganglia, trigeminal nucleus caudalis, and cortical areas (9, 10, 39, 47). The NLRP3 inflammasome, a cytosolic protein complex, upon activation, leads to the activation of proinflammatory caspase-1 (9). Activated caspase-1 fosters inflammation through several pathways: (1) it facilitates the maturation and release of proinflammatory cytokines (IL-1β and IL-18); (2) it activates caspase-3, an enzyme inducing apoptosis; and (3) it triggers pyroptosis through activating the protein gasdermin D, hence initiating neuro-inflammatory signals within the brain parenchyma (10, 48). These processes are believed to play a role in central sensitization, contributing to the onset, progression, and intensification of migraine symptoms (10). Collectively, the neuro-inflammatory response is emerging as a pivotal element in the pathophysiology of migraine, characterized by intricate interactions within the neuro-glio-vascular network. This response is partially mediated by CGRP and/or CSD, and involves oxidative stress, mitochondrial dysfunction, the assembly of NLRP3 inflammasomes, activation of microglia, and the reactivity of astrocytes.

## 5 Omega-3 fatty acid and its neuroimmunological effect

Two vital types of polyunsaturated fatty acids (PUFAs) are omega-3 and omega-6 PUFAs, named for the position of the first double bond in relation to the end methyl group. Both omega-3 and omega-6 PUFAs play significant roles in cardiovascular, inflammatory, metabolic, neurological, and psychiatric health (14, 49, 50). Omega-3 PUFAs originate from alpha-linolenic acid (ALA), while omega-6 PUFAs come from linoleic acid (LA). These are deemed essential fatty acids because they must be acquired through the diet, as the human body cannot synthesize them independently.

Alpha-linolenic acid (ALA) and linoleic acid (LA) undergo competitive metabolism through two distinct enzymatic pathways. In the liver, a sequence of desaturation, elongation, and β-oxidation reactions converts ALA into EPA and docosahexaenoic acid (DHA), while LA is transformed into AA (51). The absence of desaturase enzymes necessary for the interconversion between omega-6 and omega-3 PUFAs renders these two families of PUFAs metabolically distinct and potentially opposed in their physiological functions (52). Omega-3 PUFAs are crucial for brain development and function. DHA represents the most significant omega-3 PUFA in the brain, comprising over 40% of the brain’s total omega-3 content, especially concentrated within the gray matter (13). It plays vital roles in neurotransmission, neuroplasticity, and signal transduction (53). Specifically, DHA significantly influences the biophysical characteristics of neuronal and glial cell membranes, facilitating immunomodulatory effects (13, 54). Both DHA and EPA act as natural ligands for various nuclear receptors, including peroxisome proliferator-activated receptors, which are abundantly present in microglia and pivotal for the transcriptional regulation of many cellular activities, such as lipid balance and inflammation control (54).

### 5.1 Anti-inflammatory and anti-nociceptive effects

Omega-3 and omega-6 polyunsaturated fatty acids (PUFAs) play distinct roles in inflammation, with omega-3 PUFAs generally exhibiting anti-inflammatory effects and omega-6 PUFAs tending towards pro-inflammatory actions, through competitive metabolic processes. They act as precursors for various bioactive lipid mediators, known collectively as oxylipins (55). The production of oxylipins from tissue PUFAs depends on both the dietary intake of these fatty acids and their local availability for incorporation into cell membrane phospholipids during metabolic processing (55). Despite the liver and brain’s capacity to convert ALA into DHA, the endogenous synthesis of EPA and DHA in the brain is relatively low, necessitating their active uptake from the bloodstream (54, 56). The modern diet, which typically contains up to 15 times more omega-6 PUFAs than omega-3 PUFAs, along with a common deficiency of DHA in the brain, results in the production of large amounts of lipid mediators derived from LA and AA, contributing to chronic inflammation and oxidative stress implicated in cardiovascular, metabolic, inflammatory, and neuropsychiatric disorders (18, 50, 57–59).

PUFAs are released from cell membranes by the action of phospholipase enzymes, such as phospholipase A2 and phospholipase C, and are then converted into various bioactive compounds by cyclooxygenase, lipoxygenase, and cytochrome P450 enzymes. The predominant oxylipins include AA derivatives like prostaglandins, thromboxanes, and leukotrienes (60). EPA can be transformed into three-series prostaglandins, thromboxanes, five-series leukotrienes, endocannabinoids, eicosapentanoyl glycerol, and eicosapentaenoyl ethanolamide, while DHA gives rise to docosahexaenoyl ethanolamide and docosahexaenoyl glycerol (49). Despite structural similarities to AA-derived eicosanoids, some EPA-derived eicosanoids are potent anti-inflammatory and anti-nociceptive agents. Oxylipins regulate a broad range of physiological processes, including cell death, tissue repair, coagulation, cell proliferation, vascular permeability, pain, inflammation, immune functions, and blood pressure (55).

Specialized pro-resolving mediators (SPMs) are a class of PUFA-derived lipid mediators (49). Different SPMs are derived from differing PUFA substrates: E-series resolvins (RvE1, RvE2, and RvE3) are derived from EPA; D-series resolvins (RvD1, RvD2, RvD3, RvD4), protectins (protectin D1, a.k.a. neuroprotectin D1 when formed in the nervous system), and maresins are derived from DHA; and lipoxins are derived from AA (49, 61). SPMs are critical for resolution of inflammation, pain reduction, and tissue regeneration via specific cellular and molecular mechanisms (62). During the resolution of inflammation, SPMs produced by polymorphonuclear cells and macrophages act to limit local tissue damage caused by inflammatory responses, including signaling cessation of polymorphonuclear cell infiltration, macrophage switching to an anti-inflammatory phenotype, apoptotic cell clearance, and reducing pain caused by nociceptor sensitization (49, 61). SPMs can decrease levels of pro-inflammatory cytokines, such as IL-1β, IL-6, and tumor necrosis factor (TNF)-α, through activating their G protein-coupled receptors, thus causing activation of anti-inflammatory transcription factors, such as peroxisome proliferator-activated receptor gamma, and down-regulating intracellular NF-kB signaling pathway (14, 63). SPMs have both anti-inflammatory and pro-resolving properties without immune suppression. Although these activities are distinct, they act in tandem to promote defense from injury, inflammation, and eventually restoration of tissue homeostasis.

Astrocytes, which hold about 10–12% of the brain’s DHA, alongside oligodendrocytes and microglia, which account for 5% and 2%, respectively (64), express specific receptors for SPMs. These receptors, such as ALX/FPR2 and ChemR23/ERV1 on astrocytes, play crucial roles in controlling astrogliosis, inhibiting inflammation, and supporting neuroprotection. Microglia express a wide array of SPM receptors, whose activation encourages anti-inflammatory responses and homeostatic restoration. Thus, omega-3 PUFA-derived SPMs effectively balance the neuroinflammatory and pain processes in the interaction between glial cells and neurons (18).

### 5.2 Mitochondrial stability and antioxidant effects

Although the effects of omega-3 PUFAs on ROS production within mitochondria have yielded mixed results, omega-3 PUFAs are known to influence mitochondrial function in several ways, including impacts on membrane potential, respiration rates, the activity of mitochondrial complexes, and ROS production. These effects are linked to alterations in mitochondrial structure, such as changes in membrane phospholipid composition, viscosity, and the structure and function of lipid microdomains, which in turn affect membrane-bound signaling proteins (65, 66). Moreover, omega-3 PUFAs can enhance a cell’s endogenous antioxidant capabilities through multiple mechanisms: they can increase the activity of enzymes involved in antioxidant defense, such as glutathione peroxidase and superoxide dismutase, thus enhancing resistance to ROS-induced damage and reducing lipid peroxidation; inhibit the activity of cyclooxygenase-2 enzyme, reducing the production of pro-inflammatory and pro-oxidant prostaglandins along with lipid peroxidation; and elevate the expression of nuclear factor-erythroid 2-related factor 2 (Nrf2), a key transcription factor that governs the expression of a wide array of antioxidant and anti-inflammatory genes (65, 67–69). Therefore, omega-3 PUFAs play a critical role in supporting mitochondrial dynamics, biogenesis, structural organization, and the kinetics of mitochondrial respiration.

### 5.3 Antidepressant and anxiolytic effects

Elevated levels of systemic inflammation, neuroinflammation, and hyperactivity of the hypothalamic–pituitary–adrenal (HPA) axis have been identified as key factors contributing to the pathogenesis of neuropsychiatric disorders, including depression and anxiety (70–74). These disorders, alongside post-traumatic stress disorder, are prevalent psychiatric conditions co-occurring with migraine, significantly influencing the process of migraine becoming chronic (75, 76). Omega-3 polyunsaturated fatty acids (PUFAs) potentially modulate neurobiological pathways implicated in the coexistence of migraine with depression and/or anxiety through several mechanisms. Firstly, by dampening inflammatory responses integral to the pathophysiology of depression and anxiety, omega-3 PUFAs and their metabolites can inhibit leukocyte chemotaxis, reduce the expression of adhesion molecules, decrease the production of pro-inflammatory mediators, and encourage a shift in microglia polarization towards a reparative, non-inflammatory state (75, 76). Furthermore, they may modulate inflammatory cytokine signaling that affects glucocorticoid receptor functionality and the negative feedback control of the HPA axis; the down-regulation of inflammatory markers by omega-3 PUFAs enhances the responsiveness of the HPA-axis to negative feedback, thereby curbing HPA axis overactivity (70).

The endocannabinoid system, which is intrinsically linked to dietary lipids, neuroplasticity, and mood regulation, plays roles in synaptic plasticity, learning, memory, neuroinflammation, pain management, stress responses, and immune function (77). SPMs derived from omega-3 PUFAs have been shown to influence depressive and anxiety-related behaviors through modulation of several molecular signaling pathways, including mTOR, MAP/ERK, NF-kB, PI3K/Akt, and AMPA (14, 59). The endocannabinoids produced from arachidonic acid (AA) include anandamide and 2-arachidonoylglycerol, whereas those from omega-3 PUFAs include docosahexaenoyl ethanolamide (from DHA) and oleylethanolamide and palmitoylethanolamide (from EPA) (78). These endocannabinoids primarily target CB1 and CB2 receptors found in microglia, glial cells, and neurons (54). Endocannabinoids derived from omega-3 PUFAs serve as precursors to more potent bioactive compounds that operate via both cannabinoid-dependent and independent pathways, exerting anti-inflammatory, anti-proliferative, and mood-regulating effects (59, 79). Therefore, omega-3 PUFAs act as immunomodulatory agents that play a significant role in the regulation of inflammation and mood.

**Figure 1:**
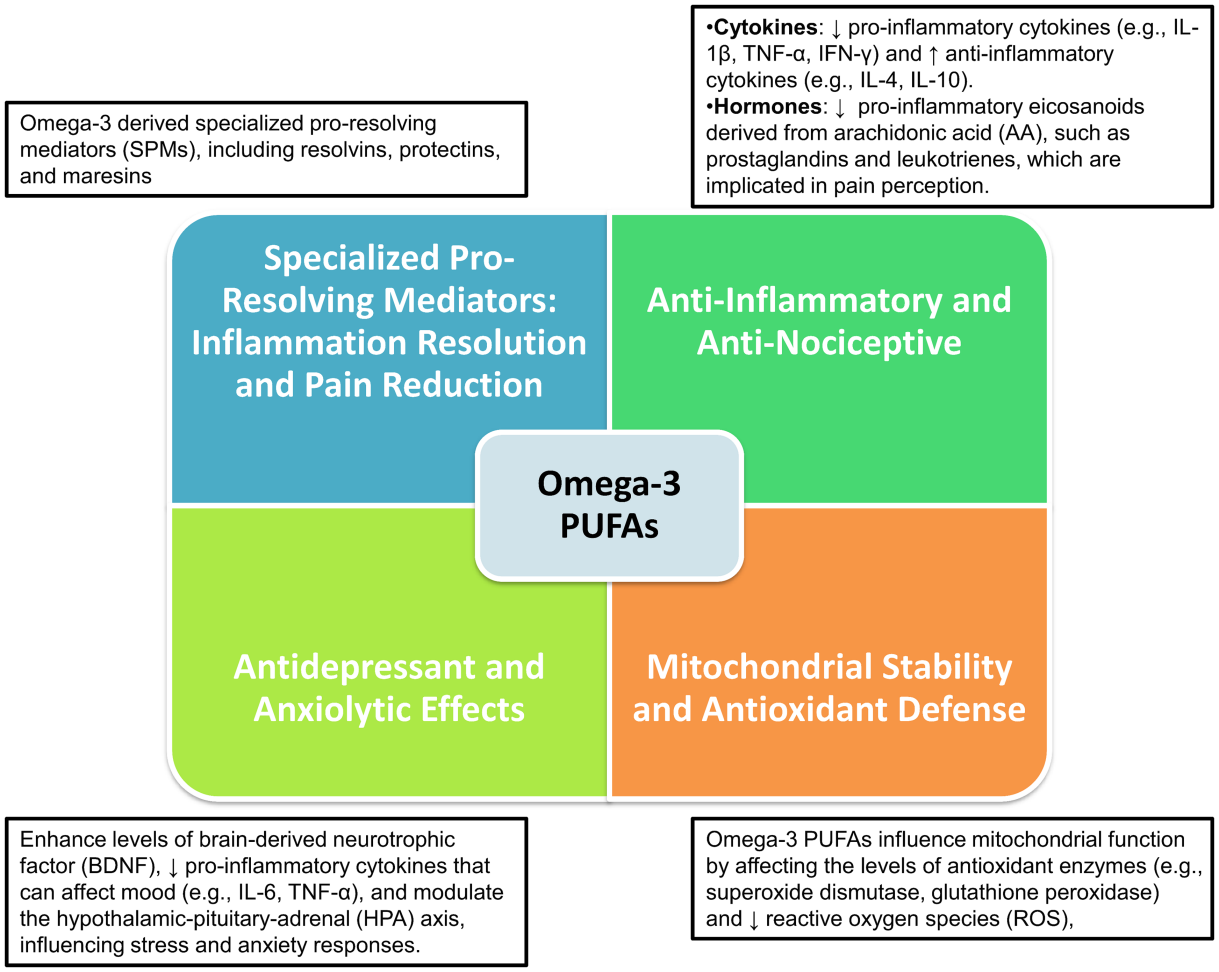
Neuropsychoimmunological impact of omega-3 PUFAs on brain functions and migraine pathophysiology.

Overall, omega-3 PUFAs and their metabolites play an essential role in neuropsychoimmunology, influencing a range of brain processes including neuroinflammation, pain signaling, mitochondrial function, oxidative stress, and mood regulation. These aspects are deeply interconnected with the underlying mechanisms of migraine pathophysiology as depicted in Figure 1.

## 6 Summary of potential benefits of omega-3 PUFAs for migraine

Omega-3 PUFAs play a pivotal role in supporting neurological health and mitigating neuroinflammation through a series of biochemical processes. These processes include influencing membrane fluidity, generating anti-inflammatory mediators, affecting intracellular signaling, and modulating gene expression (17, 80, 81). The extensive physiological actions of omega-3 PUFAs involve neurons, microglia, and astrocytes, facilitating the modulation of neuroinflammation, pain signal propagation, mitochondrial dysfunction, oxidative stress, and mood dysregulation, all of which are integral to the complex pathology of migraines (80, 81). Specifically, omega-3 PUFAs contribute to reducing CNS levels of critical inflammatory mediators like TNF-α, cyclooxygenase-2/NO synthase, and IL-1β. These reductions are believed to alleviate neuroinflammation and neurogenic pain, crucial elements in migraine pathophysiology. Moreover, supplementation with EPA and DHA has been shown to modulate nociceptive responses, potentially through activating the opioid system, offering benefits in neuropathic pain scenarios. Additionally, these fatty acids help rebalance vital neurotransmitters, such as serotonin and dopamine, essential for the functioning of the trigeminovascular nociceptive pathway, and can inhibit TNF-α expression, potentially reducing cerebral vasodilation implicated in migraine episodes. The noted high efficacy of EPA/DHA supplementation, combined with its favorable acceptability and patient compliance, sets it apart from traditional pharmacological treatments, which often exhibit higher adverse event rates and lower adherence (81). This blend of effectiveness, safety, and tolerability highlights the potential of omega-3 PUFAs in offering a more comprehensive and patient-centric approach to migraine management, thus enhancing the quality of life for those affected by this incapacitating ailment.

Omega-3 PUFAs and their metabolites, such as oxylipins and SPMs, potentially offer relief from migraines and aid in mood regulation. They achieve this by modifying the biophysical properties of neuronal and glial cell membranes, suppressing pro-inflammatory mediators, fostering the resolution of inflammation, enhancing mitochondrial stability, and reducing reactive oxygen species (ROS) production (55). Discrepancies in clinical outcomes concerning the impact of omega-3 PUFA supplementation on the frequency, severity, and duration of migraines may be attributed to methodological variations. These include differences in dosages, PUFA ratios, treatment durations, study sample sizes, and the heterogeneity of study populations, especially concerning medical and psychiatric comorbidities (17, 80, 81).

Regarding the potential short-term and long-term side effects associated with high-dose omega-3 supplementation, concerns about bleeding risks have been meticulously evaluated. However, current research suggests that daily doses of up to 4 grams are generally safe, even in conjunction with antiplatelet or anticoagulant therapies (82). Although mild gastrointestinal symptoms might occur, severe adverse effects are rare. It’s critical to recognize that PUFAs are vulnerable to lipid oxidative degradation caused by free radicals, potentially presenting health risks over extended periods (83). Presently, there’s a lack of extensive data on the long-term safety and tolerability of high-dose, prolonged omega-3 supplementation, underscoring the need for further investigation (84). Despite these considerations, the broad neuroimmunological regulatory effects of omega-3 PUFAs make them compelling candidates as therapeutic agents or adjuncts in the management of migraines.

## 7 Clinical trials of omega-3 PUFAs for migraine prevention

Omega-3 PUFAs, specifically EPA and DHA, present significant potential benefits in the prophylaxis of migraines. Emerging evidence, particularly from a comprehensive network meta-analysis (NMA), highlights that high-dosage EPA/DHA supplementation can markedly reduce the frequency and severity of migraines (85). This finding positions these omega-3 PUFAs as a promising treatment option, offering an effective alternative to the currently available pharmacological strategies which typically show limited efficacy and patient acceptability.

Djalali et al. (86) conducted a randomized, double-blind, placebo-controlled trial over 8 weeks with 40 patients prone to episodic migraines, finding that omega-3 PUFAs supplementation increased anti-inflammatory cytokine IL-4 and decreased pro-inflammatory cytokine IFN-γ levels, suggesting a potential beneficial effect on inflammatory responses in migraine patients. Rist et al. (87) utilized a two-by-two factorial design over 4.6 years with 25,871 middle-aged or older adults, including 1,032 with a history of probable migraine, and found that neither vitamin D nor marine omega-3 PUFAs supplementation affected migraine frequency or severity compared to a placebo. Honarvar et al. (88) in an 8-week randomized double-blind, placebo-controlled trial with 80 individuals experiencing episodic migraines, discovered that the combination of omega-3 PUFAs and nano-curcumin significantly reduced migraine attack frequency and serum levels of IL-1β in a synergistic manner, though the initial significant reduction in IL-1β gene expression did not remain significant after Bonferroni corrections. Soares et al. (89), through a 60-day prospective, experimental, controlled, double-blind study with 60 patients diagnosed with chronic migraine, predominantly women, observed that omega-3 PUFAs intake led to a significant reduction in headache days, indicating the benefit of omega-3 PUFAs in migraine prophylaxis. Abdolahi et al. (90) evaluated the effects of omega-3 PUFAs and nano-curcumin supplementation over 8 weeks in 80 episodic migraine patients, noting a decrease in IL-6 and hs-CRP levels, with a potential synergistic effect in the combination group. Harel et al. (91), in a 16-week randomized, double-blind, cross-over study with 27 adolescents suffering from migraines, suggested that dietary supplementation with fish oil rich in very long-chain omega-3 PUFAs could reduce the frequency, duration, and severity of migraines, a benefit also observed with olive oil. Pradalier et al. (92) conducted a 16-week randomized, double-blind, controlled trial with 196 patients, finding that omega-3 PUFAs significantly reduced the total number of migraine attacks compared to placebo, though a strong placebo effect was observed, and the study did not confirm earlier findings from smaller studies. The outcomes of these interventions have demonstrated inconsistency, with previous research predominantly focusing on mixed types of PUFAs (EPA combined with DHA) rather than examining EPA or DHA individually. Recently, Wang et al. (93) carried out a 12-week randomized, double-blind, placebo-controlled trial involving seventy individuals with episodic migraine, splitting them into two groups: 35 received 2 g of fish oil containing 1.8 g of EPA daily, while the other 35 were given 2 g of soybean oil daily as a placebo. The intervention showed significant effects, with the EPA group experiencing fewer monthly migraine days, reduced usage of headache medication, and improvements in headache severity (VAS score), disability (MIDAS score), anxiety and depression (HADS score), and quality of life (MSQ score). The main findings highlight that high-dose EPA substantially reduced migraine frequency and severity, improved psychological symptoms, and enhanced the quality of life for episodic migraine patients, suggesting its potential as an effective prophylactic, particularly for female patients.

Table 1 summarized that omega-3 fatty acids have the potential to modulate inflammatory responses and reduce migraine frequency or severity in certain populations, particularly when used in combination with other supplements like nano-curcumin. However, the effectiveness appears to be influenced by various factors including age, baseline health status, and the presence of other nutritional interventions, necessitating further research to clarify the conditions under which omega-3 supplementation might be most beneficial for migraine sufferers.

**Table 1:** The summary of the neuroimmunological effects of omega-3 fatty acids on migraine.

Study	Study design	Duration	Population characteristics	Primary outcome	Intervention effects	Main findings	Limitations
Wang et al. (93)	Randomized, double-blind, placebo-controlled trial	12 weeks	Seventy episodic migraine (EM) Total: 70 EPA group: 35 (2 g fish oil with 1.8 mg of EPA daily)—Placebo group: 35 (2 g soybean oil daily)	Decrease in migraine attacks over a 12-week period, assessed by the change in the frequency of migraine days per month from the baseline to the 12th week.	Significant reductions in Monthly migraine days: EPA group had fewer migraine days compared to placebo (*p* = 0.001). Headache medication usage days: Reduced in the EPA group (*p* = 0.035). Headache severity (VAS score), disability (MIDAS score), anxiety and depression (HADS score), and improved quality of life (MSQ score) also noted.	High-dose EPA significantly reduced migraine frequency and severity, improved psychological symptoms, and enhanced quality of life in EM patients without major adverse events. Highlights EPA’s potential as a prophylactic for EM, especially beneficial for female patients.	Single-center study with a small sample size and short treatment duration may affect generalizability. Follow-up period limited, long-term effectiveness unclear. Additional controls for dietary intake of omega-3 PUFA not accounted for. Focus on EM prevention, effects on chronic migraine not evaluated. Did not measure inflammatory biomarkers to correlate improvements with changes in inflammation. Predominantly female participants, limiting insights into efficacy in men.
Djalali et al. (86) To investigate the efficacy of omega-3 fatty acids supplementation on inflammatory and anti-inflammatory markers in patients with migraines	Randomized, double-blind, placebo-controlled trial	8 weeks	Patients prone to experiencing episodic migraines Total: 40 Omega-3 supplementation group: 20 Placebo group: 20	Change in concentrations of IL-4 and IFN-γ levels after omega-3 supplementation compared to placebo	IL-4 concentration: Omega-3 fatty acids resulted in a significant rise (*p* = 0.010) IFN-γ concentration: Omega-3 fatty acids led to a significant reduction (*p* = 0.001) TGF-β concentration: No significant change IL-17 concentration: No significant change	Omega-3 fatty acid supplementation increased anti-inflammatory cytokine IL-4 and decreased pro-inflammatory cytokine IFN-γ levels in patients with migraines, suggesting a potential beneficial effect on the inflammatory immune response.	Small sample size Short duration of the study Limited generalizability to patients with chronic migraines or other types of headaches
Rist et al. (87) To evaluate whether vitamin D supplementation and marine omega-3 (n-3) fatty acid supplementation may reduce migraine frequency or severity	Two-by-two factorial design	4.6 years	Middle-aged or older adults, Individuals with a history of probable migraine Total: 25,871 Participants with history of probable migraine: 1032	The effect of vitamin D and marine n-3 fatty acid supplementation on migraine frequency and severity.	Vitamin D: Decreases in migraine frequency: Active: 69.0% Placebo: 68.4% *p*-value: 0.54 (non-significant) Decreases in migraine severity: Active: 64.1% Placebo: 65.0% *p*-value: 0.86 (non-significant) Marine n-3 fatty acid: Decreases in migraine frequency: Active: 67.8% Placebo: 69.6% *p*-value: 0.82 (non-significant) Decreases in migraine severity: Active: 64.5% Placebo: 64.5% *p*-value: 0.96 (non-significant)	Neither vitamin D nor marine n-3 fatty acid supplementation, compared to placebo, affected migraine frequency or severity among middle-aged or older adults.	Lack of significant effects observed in migraine frequency or severity with vitamin D or n-3 fatty acid supplementation Reliance on self-reported changes in migraine frequency and severity Limited to middle-aged or older adults Potential for other unmeasured confounding variables influencing migraine outcomes
Honarvar et al. (88) To investigate the synergistic relationship between n-3 fatty acids and nano-curcumin on IL-1β gene expression and serum levels in migraine patients.	Randomized double-blind, placebo-controlled trial	8 weeks	Individuals with episodic migraines, Migraine patients Total: 80 n-3 fatty acids and curcumin combination: 20 n-3 fatty acids: 20 nano-curcumin: 20 n-3 fatty acids and curcumin placebo: 20	Reduction in attack frequency in migraine patients	n-3 fatty acids and nano-curcumin combination: Significantly reduced attack frequency in a synergistic status (*p* < 0.001) Greater reduction in serum level of IL-1β compared to other groups Significant reduction in IL-1β gene expression compared to other treatment groups (*p* < 0.05)	The combination of n-3 fatty acids and nano-curcumin significantly reduced migraine attack frequency in a synergistic manner. The combination group showed a significantly greater reduction in serum levels of IL-1β compared to other treatment groups. Although the IL-1β gene expression was significantly reduced in the combination group initially, these differences were not significant after multiple testing Bonferroni corrections.	Significant differences in IL-1β gene expression were not maintained after multiple testing Bonferroni corrections. Further studies are needed to confirm the findings.
Soares et al. (89) To determine the prophylactic effect of OPFAϖ-3 in migraine	Prospective, Experimental, Controlled, Double-blind, With comparison groups	60 days	Patients diagnosed with chronic migraine, predominantly women Total: 60 Complete: 51 OPFAϖ-3 group: 27 Placebo group: 24	Reduction in the number of days of headache per month	Reduction of more than 80.0% per month in the number of days of headache: OPFAϖ-3 group: 66.7% (18/27) Control group: 33.3% (8/24) (significant difference, χ2 = 5.649; *p* = 0.036)	Patients who used OPFAϖ-3 experienced a significant reduction in the number of headache days compared to the control group. Polyunsaturated omega 3 fatty acids (OPFAϖ-3) are beneficial for the prophylaxis of migraine attacks.	Small sample size Gender imbalance in the completed treatment group Lack of information on the reasons for dropouts Lack of information on the specific dosage of OPFAϖ-3 Lack of information on potential side effects Short duration of the study Lack of information on long-term effects
Abdolahi et al. (90) To evaluate the combined effects of ω-3 fatty acids and nano-curcumin supplementation on IL-6 gene expression, serum levels, and hs-CRP levels in migraine patients	Randomized controlled trial with four groups, measuring outcomes at the beginning and end of the study period	8 weeks	Population characteristics: episodic migraine patients Total: 80 Combination of ω-3 fatty acids (2,500 mg) plus nano-curcumin (80 mg): 20 ω-3 (2,500 mg):20 Nano-curcumin (80 mg): 20 Control (ω-3 and nano-curcumin placebo included oral paraffin oil): 20	The evaluation of the combined effects of ω-3 fatty acids and nano-curcumin supplementation on IL-6 gene expression, serum level, and hs-CRP levels in migraine patients.	Combination of ω-3 fatty acids (2,500 mg) plus nano-curcumin (80 mg): Down-regulated IL-6 mRNA Significantly decreased serum IL-6 concentration Significantly decreased hs-CRP serum levels Greater reduction of IL-6 and hs-CRP compared to nano-curcumin alone ω-3 fatty acids (2,500 mg): Down-regulated IL-6 mRNA Significantly decreased serum IL-6 concentration Nano-curcumin (80 mg): Down-regulated IL-6 mRNA Significantly decreased serum IL-6 concentration Significantly decreased hs-CRP serum levels	Both ω-3 fatty acids and nano-curcumin supplementation led to a decrease in IL-6 levels and hs-CRP levels, with a possible synergistic effect observed in the combination group.	Not mentioned
Harel et al. (91) To examine whether dietary supplementation with fish oil rich in very long-chain n-3 polyunsaturated fatty acids might reduce frequency and severity of migraines in adolescents.	randomized, double-blind, cross-over study	16 weeks	Adolescents aged around 15 years 16 girls and 7 boys Suffering from frequent migraines for at least 1 year Total: 27	Reduction in headache frequency, duration, and severity in adolescents suffering from migraines after treatment with fish oil compared to placebo (olive oil)	Frequency of headaches: Fish oil treatment: 4 +/− 1 episodes/2 months Placebo (olive oil) treatment: 4 +/− 1 episodes/2 months Headache severity: Fish oil treatment: 2.9 +/− 0.5 on a 7-point scale Placebo (olive oil) treatment: 3.5 +/− 0.4 on a 7-point scale Patients’ ratings: Fish oil treatment: Reduction in headache frequency: 87% Reduction in headache duration: 74% Reduction in headache severity: 83% Placebo (olive oil) treatment: Reduction in headache frequency: 78% Reduction in headache duration: 70% Reduction in headache severity: 65%	The main findings of the study suggest that both fish oil and olive oil may be beneficial in reducing the frequency, duration, and severity of migraines in adolescents.	Preliminary nature of the study Potential placebo effect influencing results Reliance on self-assessment by participants Small sample size
Pradalier et al. (92) To investigate the effects of Omega-3 polyunsaturated fatty acids (OPFA) on migraine prophylaxis by assessing their impact on inflammatory reactions, cytokine production, 5HT release by platelets, vasorelaxant activity, and the number of migraine attacks	Randomized, double-blind, controlled trial with intention-to-treat analysis	16 weeks	The study population characteristics include 196 patients in the intention-to-treat population, with 96 patients receiving OPFA treatment and 87 receiving a placebo. The patients were randomized and treated in a double-blind manner. Total: 196 Complete: 183 OPFA: 96 Placebo: 87	The number of migraine attacks during the last 4 weeks of treatment and the total number of attacks during the 4-month period of the study.	Mean number of migraine attacks during the last 4 weeks of treatment: OPFA group: 1.20 ± 1.40 Placebo group: 1.26 ± 1.11 (non-significant) Total number of migraine attacks during the 4-month study period: OPFA group: 5.95 Placebo group: 7.05 (significant, *p* = 0.036) No significant differences in mean intensity, mean duration of attacks, and rescue medication use. Tolerance was satisfying except for eructations where there was a significant difference against OPFA. Strong placebo effect observed with a 45% reduction in attacks between run-in and treatment period. The study did not confirm findings from two previous smaller studies.	Omega-3 polyunsaturated fatty acids (OPFA) significantly reduced the total number of migraine attacks compared to placebo, with a strong placebo effect observed in the trial. Tolerance to OPFA was generally satisfactory, except for eructations. The study did not confirm previous findings from smaller studies.	Possible bias due to the lack of confirmation of previous studies with small sample sizes, strong placebo effect observed, single-blind placebo run-in period, no significant difference in key migraine parameters, satisfying tolerance except for eructations

## 8 Future perspective

Omega-3 PUFAs could provide advantages for enhancing migraine management; however, prior randomized controlled trials, reviews, and meta-analyses have yielded inconsistent results regarding symptom improvement (17). Before omega-3 PUFAs can be endorsed for use either as a primary or supplementary option for migraine prevention, key challenges must be resolved. These include fine-tuning dosages, formulations, and the ratios of PUFAs, along with identifying the optimal durations for treatment.

Historically, omega-3 supplements were derived from fatty fish species such as salmon, mullet, and mackerel. However, increased fish demand due to the expansion of aquaculture, pressure on dwindling marine species, and marine pollution have prompted the search for alternative omega-3 PUFA sources (94). In light of these concerns, sources such as seeds, herbs, genetically engineered plants, microalgae, macroalgae, and thraustochytrids are now being investigated for the development of vegan omega-3 PUFA commercial products (83). Lipids are prone to oxidation, leading to the formation of unsaturated carbonyls and harmful by-products during extraction, storage, and processing (95). With a shift in consumer preferences towards natural products over synthetic ones, there has been an increased emphasis on incorporating natural antioxidants (94). Micro- and nano-encapsulation presents an effective strategy for shielding core PUFAs from oxygen, light, and transition metals, thereby improving their oxidative stability and bioactivities (96).

As there are no definitive markers to determine which patient groups would most benefit from this treatment method, additional research is necessary to clearly define the demographic and clinical factors linked to treatment efficacy (85). These factors include age, sex, existing comorbidities, targeted symptoms, blood biomarkers, and specific treatment protocols, before fully endorsing the use of omega-3 PUFAs either as an independent treatment or in conjunction with other medications for migraine management (80). Furthermore, conducting risk–benefit evaluations of omega-3 PUFA administration is crucial, aimed at identifying patient groups for whom omega-3 PUFA therapy would present an optimal risk–benefit balance (97).

## 9 Conclusion

The favorable impacts of omega-3 PUFAs on the neurovascular microenvironment indicate their potential to influence neural and immune cells in the brain through various physical and biochemical pathways, leading to outcomes such as anti-inflammatory, anti-nociceptive, antioxidative, and mood regulation. Omega-3 PUFAs might play a crucial role in sustaining both physical and mental health, as well as managing neuropsychiatric symptoms during the acute and chronic phases of migraines. Despite the lack of definitive evidence from well-conducted randomized trials on the effectiveness of regular daily omega-3 PUFA supplementation in mitigating migraine symptoms for those with episodic or chronic migraines, the overall positive effects in different areas (e.g., immune, neurological, psychiatric, and cardiovascular) suggest that omega-3 PUFAs could be considered as potential health supplements or adjuncts in migraine prevention and treatment. To fully explore and leverage the neuroimmunological effects of omega-3 PUFA bioactivities for migraine prevention and treatment, extensive epidemiological studies, experimental research, and randomized controlled trials are essential for thorough testing, validation, and effective implementation.

## Author contributions

T-BC: Conceptualization, Data curation, Formal analysis, Investigation, Methodology, Resources, Writing – original draft. C-CY: Conceptualization, Formal analysis, Investigation, Resources, Writing – original draft. I-JT: Conceptualization, Data curation, Formal analysis, Investigation, Methodology, Writing – original draft. H-WY: Data curation, Investigation, Writing – original draft. Y-CH: Investigation, Supervision, Writing – review & editing. C-MC: Investigation, Methodology, Project administration, Supervision, Validation, Writing – review & editing. C-PY: Conceptualization, Project administration, Supervision, Validation, Writing – review & editing.
